# Gender Differences in the Difficulty in Disengaging from Threat among Children and Adolescents With Social Anxiety

**DOI:** 10.3389/fpsyg.2017.00419

**Published:** 2017-03-24

**Authors:** Peng Zhang, Wenjin Ni, Ruibo Xie, Jiahua Xu, Xiangping Liu

**Affiliations:** ^1^School of Psychology, Beijing Normal University, BeijingChina; ^2^Faculty of Education, Beijing Normal University, BeijingChina; ^3^School of Brain and Cognitive Sciences, Beijing Normal University, BeijingChina

**Keywords:** gender differences, attentional bias, difficulty disengagement, social anxiety, adolescents

## Abstract

There is some research showing that social anxiety is related with attentional bias to threat. However, others fail to find this relationship and propose that gender differences may play a role. The aim of this study was to investigate the gender differences in the subcomponents of attentional bias to threat (hypervigilance and difficulty in disengaging) among children and adolescents with social anxiety. Overall, 181 youngsters aged between 10 and 14 participated in the current study. Images of disgusted faces were used as threat stimuli in an Exogenous Cueing Task was used to measure the subcomponents of attentional bias. Additionally, the Social Anxiety Scale for Children was used to measure social anxiety. The repeated measures ANOVA showed that male participants with high social anxiety showed difficulty in disengaging from threat, but this was not the case for female participants. Our results indicated that social anxiety is more related with attentional bias to threat among male children and adolescents than females. These findings suggested that developing gender-specific treatments for social anxiety may improve treatment effects.

## Introduction

Social anxiety is one of the most prevalent forms of anxiety, which can develop into social anxiety disorder (Costello et al., 2005). Individuals with social anxiety fear negative evaluation and persistently avoid social situations (Stein and Stein, 2008). This causes them to experience difficulty eating, communicating, and talking in public, and negatively impacts their social functioning (Stein, 1995; Zhang et al., 2016). Research has shown that about 7% of adolescents are affected by social anxiety (Chavira et al., 2004). Social anxiety reduces the academic ability of children and adolescents and places them at risk for insomnia, mood disorders, and problematic alcohol consumption in adulthood (Erwin et al., 2002; Buckner et al., 2006, 2008). Therefore, the focus on cognitive processing in children and adolescents with social anxiety is of paramount importance.

Cognitive models of emotional disorders have been supported by numerous studies that have shown that attentional bias is related to social anxiety levels in children and adolescents (Puliafico and Kendall, 2006; Roy et al., 2008; Hankin et al., 2010; Dudeney et al., 2015), as well as in adults (Roberts et al., 2010; Morrison and Heimberg, 2013; Fistikci et al., 2015; Wong and Rapee, 2016). For example, Shechner et al. (2013) examined eye movement in children and adolescents aged 8 – 17 and identified that those with anxiety disorders had attentional bias to threat. Hankin et al. (2010) used the modified dot probe task with youngsters aged 9 – 17, and reported that those with anxiety showed attentional bias to negative faces. Critically, attentional bias modification (ABM) has been proven effective in alleviating social anxiety among adolescents (Rozenman et al., 2011; Riemann et al., 2013; de Voogd et al., 2014) and adults (see meta-analysis, Cristea et al., 2015). For instance, de Voogd et al. (2014) found that visual search ABM might be beneficial in relieving attentional bias to threat and social anxiety in adolescents aged 13 – 16.

However, recent studies have failed to find significant correlation between attentional bias and the level of social anxiety in children, adolescents, and adults with clinical and subclinical anxiety (Benoit et al., 2007; Hadwin et al., 2009; Britton et al., 2012). Others report that there is no definitive evidence that ABM reduces social anxiety in adolescents (Bar-Haim et al., 2011; Eldar et al., 2012; Pergamin Hight et al., 2016) and adults (McNally et al., 2013; Bunnell et al., 2013; Heeren et al., 2015). Thus, the relationship between social anxiety and attentional bias remains unclear.

To resolve these contradicting findings, some studies proposed that gender differences may play a role. Previous studies have included male and female subjects, but the relationship between attentional bias and social anxiety may be different for males and females. Evidence has supported there is significant gender difference in attentional bias to threat among individuals with anxiety, including high trait anxiety, general anxiety, and social anxiety (Merritt et al., 2007; Tan et al., 2011; Tran et al., 2013; Kinney et al., 2016). For example, in a study involving 82 adolescents, Zhao et al. (2014) found significant positive correlation between the level of social anxiety and attentional bias to social threat for males, but not for females. This may be due to the different brain structure and emotional cognitive processing of men and women (Zhao et al., 2014; Kinney et al., 2016).

No previous study has attempted to explore the gender differences in the subcomponents of attentional bias to threat among individuals with HSA (high social anxiety). Prominent models of the attentional system suggest that attentional bias is a multifaceted construct, including at least three core components: hypervigilance, difficulty disengagement (DD), and avoidance (see review, Cisler and Koster, 2010). However, most of studies treat attentional bias as a unitary construct as specified below. Although some suggest that hypervigilance is related to social anxiety (Klumpp and Amir, 2010; MacLeod and Grafton, 2016), more amount of evidence argues that DD is the key factor contributing to social anxiety (Amir et al., 2003; Buckner et al., 2010; Heeren et al., 2011; Schofield et al., 2012; Heeren and Mcnally, 2016; Taylor et al., 2016). A recent meta-analysis showed that DD from threat is closely related to the evocation and maintenance of social anxiety, while it is not so for hypervigilance (Bantin et al., 2016).

In summary, the existence of gender differences in attentional bias to threat among individuals with social anxiety means that at least one subcomponent of attentional bias is responsible for this phenomenon. Based on substantial evidence of the close relationship between DD from threat and social anxiety, we assume that individuals with HSA show gender differences in DD from threat but not in hypervigilance. Based on Zhao et al.’s (2014) research, we hypothesize that males with HSA may have DD from threat, while females may not.

To test this hypothesis, we recruited 181 children and adolescents aged between 10 and 14 as participants. Images of disgusted faces were selected as the social threat stimuli, as they are considered to be a great threat to individuals with social anxiety and are often used by others (Heeren et al., 2015). We used the Social Anxiety Scale for Children (SASC, La Greca et al., 1988) to measure the level of social anxiety, and an Exogenous Cueing Task (ECT, Fox et al., 2001) to measure the DD from threat and hypervigilance. Children and adolescents are at the stage wherein rapid and important cognitive and social-emotional developments take place (Casey et al., 2010; Del Piero et al., 2016). These developments may impact our results; hence, we added age as covariant in the data analysis.

## Materials and Methods

### Participants

We used GPower 3.1 to compute the sample size and the number was 160 (Faul et al., 2009, statistical test: repeated measures ANOVA, within - between interaction; settings: power = 0.95; error prob = 0.05; effect size = 0.15; number of groups = 4; number of measurement = 3). Then a total of 213 students were recruited through posted advertisements and campus radio from a primary school and a middle school in Gansu Province, China. Among them, 181 participates submitted valid questionnaires and participated in the follow-up study. All participants were right-handed, aged between 10 and 14, without color blindness, and without neurological problems or history of psychological treatment.

### Measures

#### Social Anxiety Scale for Children

Social anxiety was measured using SASC (La Greca et al., 1988). The scale contains 10 items and uses a 3-point Likert scale (0 = never, 1 = sometimes, 2 = always). Scale scores range from 0 to 20, with higher scores corresponding to greater social anxiety. SASC consists of two dimensions: fear of negative evaluation (items 1, 2, 5, 6, 8, and 10) and social avoidance and distress (items 3, 4, 7, and 9). The Chinese version of SASC had good reliability and validity (α = 0.790, Li et al., 2006). In the current study, SASC was shown to have good reliability (overall, α = 0.735; fear of negative evaluation, α = 0.697; social avoidance and distress, α = 0.752).

#### Exogenous Cueing Task

**FIGURE 1:**
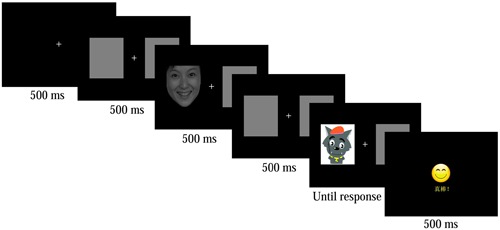
**A trial timeline of the Exogenous Cueing Task**.

This study employed the ECT used in Zhao et al.’s (2016) research. Participants started the task by pressing “Q” on the keyboard after reading and understanding the instructions. First, a fixation (“+”) was presented in the center of the computer screen. Then, a blank with two gray rectangles were shown to the right and left side of the fixation point. Next, the cue (emotional stimulus; e.g., happy face as in **Figure 1**) was presented in the left or right rectangle randomly (the other square remaining gray). The previous blank was shown again. Next, a target stimulus (either Pleasant Goat or Gray Wolf) was presented at either the location of the previous gray square (invalid position) or the cue (valid position) until the participant responded. Participants were asked to click the left mouse button (when the target stimulus was Pleasant Goat) or the right mouse button (when the target stimulus was Gray Wolf) in response to the target stimuli type. If the response was correct, a smiling face appeared and the feedback screen read “Awesome”; if not, a sad face appeared and the feedback screen read “Go for it.” Then, the next trial began. Fixation, blank, cue target stimulus and feedback were presented for 500ms. **Figure 1** shows a sample trial.

The faces with a happy, neutral, or disgust emotional expression were selected from the Chinese Affective Picture System. The proportion of male and female faces was equally distributed. The faces with different emotions did not significantly differ on an intensity dimension using a scale from 1 (lowest) – 9 (highest), *F*(2,23) = 0.700, *p* = 0.508, η^2^ = 0.062. Pleasant Goat and Gray Wolf are popular cartoons among children and adolescents in China. A 1 - 7 score (from unpleasant to pleasant) from 20 adolescents showed no significant difference in their emotional valence; *T* = -0.176, *p* = 0.861.

The practice stage involved 12 trials using neutral face cues. Participants’ accuracy was required to be higher than 90% before they began the formal experiment. Each participant had three chances to complete the practice stage. The experiment involved a total of 96 trials (2 Positions × 2 Goat or Wolf presentations × 3 cue types × 8 repetitions). The trials were randomly assigned to one of four 24-trial blocks. Participants were able to take a break between blocks and then resume the experiment by pressing “Q.” The task lasted about 15 min. A 17 cm display device with a screen resolution of 1366 × 768 and a refresh rate of 85 Hz was used to present the stimuli. Participants were placed approximately 60 cm from the screen.

If a participant’s reaction time (RT) to neutral cues was shorter than the RT for emotional cues (happy and disgusted), in response to a target stimulus in the invalid position, the participant was considered to display difficulty in disengaging from emotional cues. If a participant’s RT to neutral cues was longer than the RT for emotional cues, in response to a target stimulus in the valid position, the participant was considered to display hypervigilance for emotional cues (Fox et al., 2001).

### Procedure

The Beijing Normal University Ethics Committee approved all stages of this study. All participants provided written informed consent from themselves and their parents or legal guardians. Afterward, the participants were guided into the behavioral psychology laboratory where they completed the SASC using paper and a pencil, and then the modified ECT. The entire procedure lasted a maximum of 30 min.

## Results

### Data Reduction

Thirteen participants were eliminated from the data analysis for the following reasons: (a) three failed to reach an accuracy of 90%; (b) three had trials for which RTs lesser than 200 ms or more than 2000 ms accounted for more than 30% of the total trials; (c) two quit the experimental task because they failed to pass the practice stage; (d) two missed the experiment because of time conflicts; (e) three quit the ECT. The final sample size was 168 participants out of the 181, with a loss rate of 7.18%. The accuracy of responses for all participants was higher than 90%. We excluded error responses and outlier trials (RT > 2000 ms or < 200 ms) for RT data analysis (Zhao et al., 2016).

### Data Analysis

Data were processed using SPSS 19.0 (IBM Corporation, Armonk, NY, USA). Firstly, the independent sample *T*-test was performed to describe the differences in age, years of education, and social anxiety between males and females. Secondly, the normality tests and homogeneity of variance tests of RT showed that the data was suitable for analysis of variance. Finally, we used a median split at social anxiety score = 7 to define a LSA (low social anxiety, *N* = 92, range 0–7) and HSA group (*N* = 76, range 8–18, Mcgrath et al., 2016). Then, a Group (LSA and HSA) × Gender (male and female) × Cue Type (happy, disgusted, neutral) ANOVA on the RT was examined for the valid position (to assess hypervigilance) and invalid position (to assess disengagement) separately^1^.

### Repeated Measures ANOVA for RT

**FIGURE 2:**
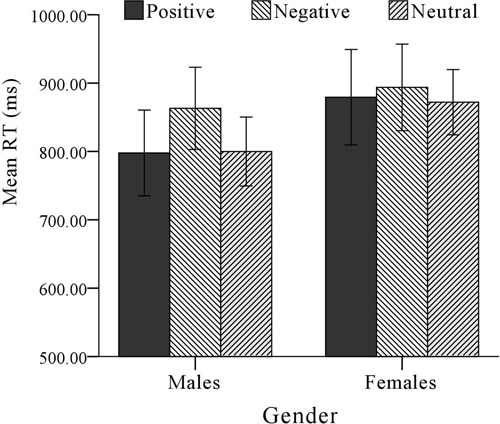
**Mean RTs on invalid position (difficulty in disengaging) during the ECT for participants with HSA**.

All RTs in each condition are shown in **Table 1**. The Group × Gender × Cue Type ANOVA on the RT for valid position showed no significant interaction and main effect (*F* < 2.830, *p* > 0.060, η^2^< 0.037). It revealed that participants had no hypervigilance to threat irrespective of social anxiety and gender. Then this three-term interaction on the RT for invalid position showed a significant interaction between Group and Gender, *F*(1,164) = 4.267, *p* = 0.040, η^2^ = 0.025. There was no other significant main effect or interactions (*F* < 2.965, *p* > 0.087, η^2^< 0.018). To further examine gender differences in DD, we conducted the Group × Cue Type ANOVA for males and females separately. Males and females in the current study did not differ in age, years of education, and social anxiety (see **Table 2**). For females, the main effect of Group was not significant, *F*(1,74) = 1.724, *p* = 0.193, η^2^ = 0.023, and the main effect of Cue Type was also not significant, *F*(2,148) = 0.386, *p* = 0.680, η^2^ = 0.005. The interaction was not significant as well, *F*(2,148) = 0.126, *p* = 0.882, η^2^ = 0.002. This revealed that female participants have not shown difficulty disengaging from threat. For males, the main effect of Group was not significant, *F*(1,90) = 2.661, *p* = 0.106, η^2^ = 0.029 and the main effect of Cue Type was also not significant, *F*(2,148) = 0.386, *p* = 0.680, η^2^ = 0.005. However, the interaction was significant, *F*(2,180) = 4.444, *p* = 0.013, η^2^ = 0.047. Simple effect analyses revealed that participants with LSA showed no differences in RT for cue types, *F*(1,90) = 2.180, *p* = 0.119, η^2^ = 0.047, while participants with HSA showed significant difference, *F*(1,90) = 2.180, *p* = 0.119, η^2^ = 0.047. Further *post hoc* tests showed that, relative to the neutral faces (*M* = 799 ms), participants with HSA responded significantly slower to disgusted faces (*M* = 863 ms, *p* = 0.033) than to happy faces (*M* = 798 ms, *p* = 0.915, see **Figure 2**). This revealed that male participants with HSA showed difficulty disengaging from threat, while this was not the case for participants with LSA.

**Table 1:** Reaction time (RT) performance in Exogenous Cueing Task (*n* = 168).

		Valid						Invalid					
		Positive		Negative		Neutral		Positive		Negative		Neutral	
		*M*	*SD*	*M*	*SD*	*M*	*SD*	*M*	*SD*	*M*	*SD*	*M*	*SD*
Male	LSA (*n* = 53)	869	162	838	181	873	195	901	205	855	180	875	202
	HSA (*n* = 39)	838	170	808	179	870	209	798	196	863	188	799	158
	Total (*n* = 92)	856	166	825	180	872	199	857	207	858	182	843	206
Female	LSA (*n* = 39)	845	146	830	175	853	201	844	177	838	169	827	177
	HSA (*n* = 37)	891	168	871	154	876	141	879	212	894	193	872	145
	Total (*n* = 76)	867	158	850	165	864	173	861	195	865	182	849	163

**Table 2:** Gender differences in ages, education years and social anxiety.

	Total (*n* = 168)		Male (*n* = 92)		Female (*n* = 76)		*t*	*p*
	*M*	*SD*	*M*	*SD*	*M*	*SD*		
Ages	11.98	1.38	11.97	1.46	12.00	1.27	-0.153	0.878
Education years	5.06	0.85	5.01	0.85	5.13	0.85	-0.910	0.364
Social anxiety	7.09	3.59	7.15	3.58	7.07	3.63	0.145	0.885

Meanwhile, we added age as a covariate in all of the above ANOVA analyses. The results of the three-term ANOVA showed that the main effects of age are significant, *F*(1,163) = 20.356, *p* < 0.001, η^2^ = 0.111), and the response of older subjects was significantly faster than that of younger subjects. However, no interaction between age and the variables was found (*F* < 1). Similar results were found in the Group × Cue Type ANOVA for males and females separately^2^.

## Discussion

In the current study we investigated the gender differences in the subcomponents of attentional bias to threat among children and adolescents with social anxiety, using the exogenous cueing task. We found that participants with HSA had no significant attentional bias to threat. The further data analysis showed that male participants with HSA showed difficulty in disengaging from social threat, while the same was not true of female participants. There was no obviously attentional bias among participants with LSA. No significant attentional bias to happy faces was found in overall participants.

Firstly, our study failed to find hypervigilance and DD among participants with HSA. This is inconsistent with the previous studies. Hypervigilance and DD are the two core components of early attentional bias. Some researchers found that individuals with HSA are hypervigilant to threat, and others believe that they have DD from threat (Heeren and Mcnally, 2016). This may be because the participants are subclinical and exhibit insensitivity to the measurement. However, this result does not take gender differences into account.

Secondly, consistent with our hypothesis, when the data were processed separately for males and females, the results indicated that males with HSA showed DD from threat, while females did not. This supports the findings of Zhao et al. (2014). They found that there was a gender difference in the relationship between attentional bias and social anxiety, and we further showed that this is due to the gender difference in DD from threat. Several studies have supported the fact that there are significant gender differences in the relationship between attentional bias and emotional processing (Merritt et al., 2007; Tan et al., 2011; Tran et al., 2013; Kinney et al., 2016), but they do not focus on the relationship between social anxiety and a subcomponent of attentional bias to threat. Our results provide further evidences for gender differences in attentional bias among socially anxious individuals.

Finally, these findings suggest that social anxiety is more related with attentional bias to threat among male children and adolescents than females. Several studies have found that men display a larger physiological response to threat than women (see review; Kudielka and Kirschbaum, 2005). This implies that men are more likely to experience anxiety compared to women when facing social threats. Our study supports this point. Moreover, there is self-reporting evidence that women tend to avoid or distract themselves from threat situations more than men (see review; Nolen-Hoeksema, 2012). This avoidance strategy may contribute to experience less anxiety. But some studies found that avoidance and safety behaviors are not always beneficial and may lead to more anxiety (Salkovskis, 1991; Clark and Wells, 1995). The effects of avoidance on anxiety need further exploring.

The present study has some limitations. First, the sample was of a limited age range (10–14 years old) and from the general population, which limits the generalization from extending to clinical samples with social anxiety and to other age groups. Second, we employed behavioral experiments, but did not examine eye movements or use brain imaging technology; these methodologies could be employed in future studies. Moreover, the relationship between cognitive bias and social anxiety is complex. It could be influenced by multiple factors, such as individual differences among participants, involvement of distinct visuals, and present time of stimuli (Holmes et al., 2005; Cooper and Langton, 2006; Massar et al., 2011). Future research could examine gender differences in the relationship between attentional bias and social anxiety employing variously threatening stimuli (such as words and videos) at both short and long stimulus durations.

## Conclusion

Attention selection system is becoming a hot topic, which has a close relationship with dysfunctional cognitive and emotional processing (Miloff et al., 2015). We found that male adolescents with HSA showed DD from threat, while the same was not true for females. This indicated that the attentional bias of children and adolescents with social anxiety may be affected by gender differences. Therefore, we should develop gender-specific treatments for social anxiety, especially in children and adolescents who have high prevalence of social anxiety. For example, focusing on improving disengagement from threat in men with social anxiety may improve the treatment response rates.

## Author Contributions

PZ and XL designed research and analyzed data; PZ and JX collected the data. PZ, WN, and RX wrote the paper. All authors contributed and have approved the final manuscript.

## Conflict of Interest Statement

The authors declare that the research was conducted in the absence of any commercial or financial relationships that could be construed as a potential conflict of interest. The reviewer VL and the handling Editor declared their shared affiliation, and the handling Editor states that the process nevertheless met the standards of a fair and objective review.

## Footnotes

1 The Group × Gender × Cue Type × Position ANOVA on the RT was conducted in the first. The four-term interaction was not significant, *F*(2,328) = 1.174, *p* = 0.311, η^2^ = 0.007. The interaction between Group and Gender was significant, *F*(1,164) = 3.294, *p* = 0.051, η^2^ = 0.032, as well as the Cue Type × Position interaction, *F*(2,328) = 3.371, *p* = 0.036, η^2^ = 0.020. Then the three-term ANOVA was examined for the valid position (to assess hypervigilance) and invalid position (to assess disengagement) separately.

2 The interaction between Age and Cue Type was not significant, for males: *F* < 1; for female: *F*(2,146) = 1.015, *p* = 0.365, η^2^ = 0.014. The main effect of age was significant, for males: *F*(1,89) = 9.022, *p* = 0.003, η^2^ = 0.092; for female: *F*(1,73) = 12.249, *p* = 0.001, η^2^ = 0.144.

## References

[B1] Amir N. Elias J. Klumpp H. Przeworski A. (2003). Attentional bias to threat in social phobia: facilitated processing of threat or difficulty disengaging attention from threat? *Behav. Res. Ther.* 41 1325–1335. 10.1016/S0005-7967(03)00039-1 14527531

[B2] Bantin T. Stevens S. Gerlash A. L. Hermann C. (2016). What does the facial dot-probe task tell us about attentional processes in social anxiety? A systematic review. *J. Behav. Ther. Exp. Psychiatry* 50 40–51. 10.1016/j.jbtep.2015.04.009 26042381

[B3] Bar-Haim Y. Morag I. Glickman S. (2011). Training anxious children to disengage attention from threat: a randomized controlled trial. *J. Child Psychol. Psychiatry* 52 861–869. 10.1111/j.1469-7610.2011.02368.x 21250993

[B4] Benoit K. E. McNally R. J. Rapee R. M. Gamble A. L. Wiseman A. L. (2007). Processing of emotional faces in children and adolescents with anxiety disorders. *Behav. Change* 24 183–194. 10.1375/bech.24.4.183

[B5] Britton J. C. Bar-Haim Y. Carver F. W. Holroyd T. Norcross M. A. Detloff A. (2012). Isolating neural components of threat bias in pediatric anxiety. *J. Child Psychol. Psychiatry* 53 678–686. 10.1111/j.1469-7610.2011.02503.x 22136196 PMC3354023

[B6] Buckner J. D. Bernert R. A. Cromer K. R. Joiner T. E. J. Schmidt N. B. (2008). Social anxiety and insomnia: the mediating role of depressive symptoms. *Depress Anxiety* 25 124–130. 10.1002/da.20282 17340615

[B7] Buckner J. D. Eggleston A. M. Schmidt N. B. (2006). Social anxiety and problematic alcohol consumption: the mediating role of drinking motives and situations. *Behav. Ther.* 37 381–391. 10.1016/j.beth.2006.02.007 17071215

[B8] Buckner J. D. Maner J. K. Schmidt N. B. (2010). Difficulty disengaging attention from social threat in social anxiety. *Cogn. Ther. Res.* 34 99–105. 10.1007/s10608-008-9205-y PMC2826845 20182655

[B9] Bunnell B. E. Beidel D. C. Mesa F. (2013). A randomized trial of attention training for generalized social phobia: Does attention training change social behavior? *Behav. Ther.* 44 662–673. 10.1016/j.beth.2013.04.010 24094791

[B10] Casey B. J. Jones R. M. Levita L. Libby V. Pattwell S. S. Rubbery E. J. (2010). The storm and stress of adolescence: insights from human imaging and mouse genetics. *Dev. Psychobiol.* 52 225–235. 10.1002/dev.20447 20222060 PMC2850961

[B11] Chavira D. A. Stein M. B. Bailey K. Stein M. T. (2004). Child anxiety in primary care: prevalent but untreated. *Depression Anxiety* 20 155–164. 10.1002/da.20039 15643639

[B12] Cisler J. M. Koster E. H. (2010). Mechanisms of attentional biases towards threat in anxiety disorders: an integrative review. *Clin. Psychol. Rev.* 30 203–216. 10.1016/j.cpr.2009.11.003 20005616 PMC2814889

[B13] Clark D. M. Wells A. (1995). *Social Phobia: Diagnosis, Assessment, and Treatment.* New York, NY: The Guilford Press.

[B14] Cooper R. M. Langton S. R. H. (2006). Attentional bias to angry faces using the dot-probe task? It depends when you look for it. *Behav. Res. Ther.* 44 1321–1329. 10.1016/j.brat.2005.10.004 16321361

[B15] Costello E. Egger H. Angold A. (2005). 10-year research update review: the epidemiology of child and adolescent psychiatric disorders: I. Methods and public health burden. *J. Am. Acad. Child Adolesc. Psychiatry* 44 972–986. 10.1097/01.chi.0000172552.41596.6f 16175102

[B16] Cristea I. A. Kok R. N. Cuijpers P. (2015). The efficacy of cognitive bias modification interventions in anxiety and depression: a meta-analysis. *Br. J. Psychiatry* 206 7–16. 10.1192/bjp.bp.114.146761 25561486

[B17] de Voogd E. L. Wiers R. W. Prins P. J. M. Salemink E. (2014). Visual search attentional bias modification reduced social phobia in adolescents. *J. Behav. Ther. Exp. Psychiatry* 45 252–259. 10.1016/j.jbtep.2013.11.006 24361543

[B18] Del Piero L. B. Saxbe D. E. Margolin G. (2016). Basic emotion processing and the adolescent brain: task demands, analytic approaches, and trajectories of changes. *Dev. Cogn. Neurosci.* 19 174–189. 10.1016/j.dcn.2016.03.005 27038840 PMC4912905

[B19] Dudeney J. Sharpe L. Hunt C. (2015). Attentional bias towards threatening stimuli in children with anxiety: a meta-analysis. *Clin. Psychol. Rev.* 40 66–75. 10.1016/j.cpr.2015.05.007 26071667

[B20] Eldar S. Apter A. Lotan D. Perez-Edgar K. Naim R. Fox N. A. (2012). Attention bias modification treatment for pediatric anxiety disorders: a randomized controlled trial. *Am. J. Psychiatry* 169 213–220. 10.1016/j.ypsy.2012.06.009 22423353 PMC3491316

[B21] Erwin B. A. Heimberg R. G. Juster H. Mindlin M. (2002). Comorbid anxiety and mood disorders among persons with social anxiety disorder. *Behav. Res. Ther.* 40 19–35. 10.1016/s0005-7967(00)00114-5 11762424

[B22] Faul F. Erdfelder E. Buchner A. Lang A. G. (2009). Statistical power analyses using G*Power 3.1: tests for correlation and regression analyses. *Behav. Res. Methods* 41 1149–1160. 10.3758/brm.41.4.1149 19897823

[B23] Fistikci N. Saatcioglu O. Keyvan A. Topcuoglu V. (2015). Attentional bias and training in social anxiety disorder. *Archiv. Neuropsychiatry* 52 4–7. 10.5152/npa.2015.8777 PMC5352998 28360667

[B24] Fox E. Russo R. Bowles R. Dutton K. (2001). Do threatening stimuli draw or hold visual attention in subclinical anxiety? *J. Exp. Psychol. Gen.* 130 681–700. 10.1037//0096-3445.130.4.681 11757875 PMC1924776

[B25] Hadwin J. A. Donnelly N. Richards A. French C. C. Patel U. (2009). Childhood anxiety and attention to emotion faces in a modified stroop task. *Br. J. Dev. Psychol.* 27 487–494. 10.1348/026151008x315503 19998543

[B26] Hankin B. L. Gibb B. E. Abela J. R. Z. Flory K. (2010). Selective attention to affective stimuli and clinical depression among youths: role of anxiety and specificity of emotion. *J. Abnorm. Psychol.* 119 491–501. 10.1037/a0019609 20677838 PMC2946390

[B27] Heeren A. Lievens L. Philippot P. (2011). How does attention training work in social phobia: disengagement from threat or re-engagement to non-threat? *J. Anxiety Disorders* 25 1108–1115. 10.1016/j.janxdis.2011.08.001 21907539

[B28] Heeren A. Mcnally R. J. (2016). An integrative network approach to social anxiety disorder: the complex dynamic interplay among attentional bias for threat, attentional control, and symptoms. *J. Anxiety Disord.* 42 95–104. 10.1016/j.janxdis.2016.06.009 27395806

[B29] Heeren A. Mogoaşe C. Philippot P. McNally R. J. (2015). Attention bias modification for social anxiety: a systematic review and meta-analysis. *Clin. Psychol. Rev.* 40 76–90. 10.1016/j.cpr.2015.06.001 26080314

[B30] Holmes A. Green S. Vuilleumier P. (2005). The involvement of distinct visual channels in rapid attention towards fearful facial expressions. *Cogn. Emot.* 19 899–922. 10.1080/02699930441000454

[B31] Kinney K. L. Boffa J. W. Amir N. (2016). Gender difference in attentional bias toward negative and positive stimuli in generalized anxiety disorder. *Behav. Ther.* (in press) 10.1016/j.beth.2016.06.002 PMC12994363 28390492

[B32] Klumpp H. Amir N. (2010). Preliminary study of attention training to threat and neutral faces on anxious reactivity to a social stressor in social anxiety. *Cogn. Ther. Res.* 34 263–271. 10.1007/s10608-009-9251-0

[B33] Kudielka B. M. Kirschbaum C. (2005). Sex differences in HPA axis responses to stress: a review. *Biol. Psychol.* 69 113–132. 10.1016/j.biopsycho.2004.11.009 15740829

[B34] La Greca A. M. Dandes S. K. Wick P. Shaw K. Stone W. L. (1988). Development of the social anxiety scale for children: reliability and concurrent validity. *J. Clin. Child Psychol.* 17 84–89. 10.1207/s15374424jccp1701_11

[B35] Li F. Su L. Y. Jin Y. (2006). Norm of the screen for child social anxiety relatedemotional disorders in Chinese urban children. *Chin. J. Child Health Care* 14 5–9.

[B36] MacLeod C. Grafton B. (2016). Anxiety-linked attentional bias and its modification: illustrating the importance of distinguishing processes and procedures in experimental psychopathology research. *Behav. Res. Ther.* 86 68–86. 10.1016/j.brat.2016.07.005 27461003

[B37] Massar S. A. A. Mol N. M. Kenemans J. L. Baas J. M. P. (2011). Attentional bias in high-and low-anxious individuals: evidence for threat-induced effects on engagement and disengagement. *Cogn. Emot.* 25 805–817. 10.1080/02699931.2010.515065 21824022

[B38] Mcgrath L. M. Oates J. M. Dai Y. G. Dodd H. F. Waxler J. Clements C. C. (2016). Attention bias to emotional faces varies by IQ and anxiety in williams syndrome. *J. Autism Dev. Disord.* 46 2174–2185. 10.1007/s10803-016-2748-y 26886469 PMC4860354

[B39] McNally R. J. Enock P. M. Tsai C. Tousian M. (2013). Attention bias modification for reducing speech anxiety. *Behav. Res. Ther.* 51 882–888. 10.1016/j.brat.2013.10.001 24211339

[B40] Merritt P. Hirshman E. Wharton W. Stangl B. Devlin J. Lenz A. (2007). Evidence for gender differences in visual selective attention. *Pers. Individ. Dif.* 43 597–609. 10.1016/j.paid.2007.01.016

[B41] Miloff A. Savva A. Carlbring P. (2015). Cognitive bias measurement and social anxiety disorder: correlating self-report data and attentional bias. *Internet Interv.* 2 227–234. 10.1016/j.invent.2015.03.006

[B42] Morrison A. S. Heimberg R. G. (2013). Social anxiety and social anxiety disorder. *Annu. Rev. Clin. Psychol.* 9 249–274. 10.1146/annurev-clinpsy-050212-185631 23537485

[B43] Nolen-Hoeksema S. (2012). Emotion regulation and psychopathology: the role of gender. *Annu. Rev. Clin. Psychol.* 8 161–187. 10.1146/annurev-clinpsy-032511-143109 22035243

[B44] Pergamin Hight L. Pine D. S. Fox N. A. Bar-Haim Y. (2016). Attention bias modification for youth with social anxiety disorder. *J. Child Psychol. Psychiatry* 57 1317–1325. 10.1111/jcpp.12599 27435286

[B45] Puliafico A. C. Kendall P. C. (2006). Threat-related attentional bias in anxious youth: a review. *Clin. Child Fam. Psychol. Rev.* 9 1 62–180. 10.1007/s10567-006-0009-x 17053961

[B46] Riemann B. C. Kuckertz J. M. Rozenman M. Weersing V. R. Amir N. (2013). Augmentation of youth cognitive behavioral and pharmacological interventions with attention modification: a preliminary investigation. *Depress Anxiety* 30 822–828. 10.1002/da.22127 23658147 PMC4005412

[B47] Roberts K. E. Hart T. A. Eastwood J. D. (2010). Attentional biases to social and health threat words in individuals with and without high social anxiety or depression. *Cogn. Ther. Res.* 34 388–399. 10.1007/s10608-009-9245-y

[B48] Roy A. K. Vasa R. A. Bruck M. Mogg K. Bradley B. P. Sweeney M. (2008). Attention bias toward threat in pediatric anxiety disorders. *J. Am. Acad. Child Adolesc. Psychiatry* 47 1189–1196. 10.1097/chi.0b013e3181825ace 18698266 PMC2783849

[B49] Rozenman M. Weersing V. R. Amir N. (2011). A case series of attention modification in clinically anxious youths. *Behav. Res. Ther.* 49 324–330. 10.1016/j.brat.2011.02.007 21444066 PMC4005407

[B50] Salkovskis P. M. (1991). The importance of behaviour in the maintenance of anxiety and panic: a cognitive account. *Behav. Psychother.* 19 6–19. 10.1017/s0141347300011472

[B51] Schofield C. A. Johnson A. L. Inhoff A. W. Coles M. E. (2012). Social anxiety and difficulty disengaging threat: evidence from eye-tracking. *Cogn. Emot.* 26 300–311. 10.1080/02699931.2011.602050 21970428 PMC3408005

[B52] Shechner T. Jarcho J. M. Britton J. C. Leibenluft E. Pine D. S. Nelson E. E. (2013). Attention bias of anxious youth during extended exposure of emotional face pairs: an eye-tracking study. *Depress Anxiety* 30 14–21. 10.1002/da.21986 22815254 PMC3541440

[B53] Stein M. B. (1995). *Social Phobia: Clinical and Research Perspectives.* Washington, DC: American Psychiatric Press.

[B54] Stein M. B. Stein D. J. (2008). Social anxiety disorder. *Lancet* 77 1115–1125. 10.1016/s0140-6736(08)60488-2 18374843

[B55] Tan J. Ma Z. Gao X. Wu Y. Fang F. (2011). Gender difference of unconscious attentional bias in high trait anxiety individuals. *PLoS ONE* 6:e20305 10.1371/journal.pone.0020305 PMC3101250 21647221

[B56] Taylor C. T. Cross K. Amir N. (2016). Attentional control moderates the relationship between social anxiety and attentional disengagement from threatening information. *J. Behav. Ther. Exp. Psychiatry* 50 68–76. 10.1016/j.jbtep.2015.05.008 26072705 PMC4656135

[B57] Tran U. S. Lamplmayr E. Pintzinger N. M. Pfabigan D. M. (2013). Happy and angry faces: subclinical levels of anxiety are differentially related to attentional biases in men and women. *J. Res. Pers.* 47 390–397. 10.1016/j.jrp.2013.03.007

[B58] Wong Q. J. J. Rapee R. M. (2016). The aetiology and maintenance of social anxiety disorder: a synthesis of complimentary theoretical models and formulation of a new integrated model. *J. Affect. Disord.* 203 84–100. 10.3389/fpsyg.2016.01963 27280967

[B59] Zhang P. Deng Y. Yu X. Zhao X. Liu X. (2016). Social anxiety, stress type, and conformity among adolescents. *Front. Psychol.* 7:760 10.3389/fpsyg.2016.00760 PMC4873518 27242649

[B60] Zhao X. Zhang P. Chen L. Zhou R. (2014). Gender differences in the relationship between attentional bias to threat and social anxiety in adolescents. *Pers. Individ. Dif.* 71 108–112. 10.1016/j.paid.2014.07.023

[B61] Zhao X. Zhang P. Fu L. Maes J. H. R. (2016). Attentional biases to faces expressing disgust in children with autism spectrum disorders: an exploratory study. *Sci. Rep.* 6:19381 10.1038/srep19381 PMC4725836 26758779

